# Empathy Enhancement Based on a Semiotics Training Program: A Longitudinal Study in Peruvian Medical Students

**DOI:** 10.3389/fpsyg.2020.567663

**Published:** 2020-10-29

**Authors:** Lissett J. Fernández-Rodríguez, Víctor H. Bardales-Zuta, Montserrat San-Martín, Roberto C. Delgado Bolton, Luis Vivanco

**Affiliations:** ^1^Faculty of Medicine, Antenor Orrego Private University, Trujillo, Peru; ^2^Faculty of Social Sciences of Melilla, University of Granada, Melilla, Spain; ^3^Department of Diagnostic Imaging (Radiology) and Nuclear Medicine, University Hospital San Pedro, Logroño, Spain; ^4^Platform of Bioethics and Medical Education, Centre for Biomedical Research of La Rioja (CIBIR), Logroño, Spain; ^5^National Centre of Documentation on Bioethics, Rioja Health Foundation, Logro o, Spain

**Keywords:** medicine students, professionalism, medical curriculum, medical semiotics, lifelong learning, teamwork ability, empathy

## Abstract

**Background:**

Empathy, as a core element of medical professionalism, is part of leadership in medicine. This attribute, predominantly cognitive, involves understanding and communication capacity. Empathy can be enhanced with courses on medical semiotics. It appears adequate to apply this enhancement in the early stages of professional training. Based on this, this study was performed with the purpose of demonstrating the positive effect that an academic course on medical semiotics has on the development of empathy in medical students.

**Methods:**

A quasi-experimental study was conducted in one School of Medicine in Peru, where medical students had to attend a 17-week course on medical semiotics as part of their regular training. The sample, composed by 269 students, included two cohorts of third-year medical students. As main measures, the Jefferson Scales of Empathy (JSE), inter-professional collaboration (JSAPNC), and lifelong learning (JeffSPLL), were used. In addition, students’ scores evaluating theoretical and practical aspects of the course were collected once the course was finished. Pre- and post-tests were administered in week 1 and in week 17. Analyses compared measures in both moments and in time. Inter-professional collaboration and lifelong learning scores and empathy scores were used as discriminant and convergent validity measures of students’ course scores, respectively.

**Results:**

Gender differences on empathy appeared, but only at the beginning. In the entire sample, empathy enhancement was confirmed in time (*p* < 0.001), with a large effect size (*r* = 0.45). This effect was also observed in both gender groups, separately. On the contrary, no changes appeared in inter-professional collaboration and in lifelong learning abilities in time. In addition, a positive correlation was observed among empathy, inter-professional collaboration and lifelong learning abilities at the beginning and at the end, confirming that the improvement observed was specific for empathy and explained by the educational intervention assessed.

**Conclusion:**

These findings bring empiric evidence supporting the positive effect that training in medical semiotics has on empathy. In addition, these findings highlight some gender differences in the development of empathy in medical students.

## Introduction

### Empathy in the Context of Emotional Intelligence and Leadership in Medicine

Empathy has been described as an important component of medical professionalism (Veloski and Hojat, 2006), and leadership in medicine (Hojat et al., 2015). Several studies have provided empirical support of the important role that empathy plays in health care linking it with positive clinical outcomes, quality of medical care, patients’ satisfaction and wellness at the workplace for healthcare professionals (Decety, 2020). This ability, in the specific context of clinical encounters, has been defined as a predominantly cognitive (rather than affective or emotional) professional competence. Three elements usually emerge as main components of empathy (Hojat, 2016, p. 74; Decety, 2020, p. 565): (i) understanding of patients’ experiences, concerns and perspectives; (ii) a good and clear communication with the patient; and (iii) an intention to help, expressed in a benevolent (compassionate) attitude intending to care a person in need. The first two elements, usually presented as “see with the mind’s eye” (understanding) and “hear with the third ear” (listening), are controlled by complex cognitive and neural processes. In the last years, a number of experimental studies have linked the cognitive and neural mechanisms associated with the “mind’s eye” with some important socioemotional processes, including social knowledge, social perception, and decision making (Hassabis et al., 2007; Wilson et al., 2020). On the other hand, the “third ear,” a concept originally coined in psychoanalysis, has been used in medicine to describe the attribute of hearing not only what the patient’s words do say but what the words do not say as well (Reik, 1948, p. 144; Good, 1972). In relation with the third element, intention to help, some authors suggest that this component is the main one responsible of emotion regulation, also described as emotional intelligence (Goleman, 1995), in healthcare professionals by controlling the personal distress that can be derived from the exposition to patients’ suffering (Decety and Jackson, 2004; Pokorski et al., 2013). In other words, keeping an intention to help as the main personal motivation allows healthcare professionals to recognize the patient as like self while maintaining a clear separation between self and the patient. Neuroimaging studies suggest that behind this emotional regulation there is a neural control of brain regions involved in emotional responses, such as the insula, the anterior cingulate cortex, and the periaqueductal gray (Cheng et al., 2007; Smith et al., 2018; Kogler et al., 2020).

### Enhancement of Empathy and Gender-Related Differences

It has been described that empathy in medical students can improve, can deteriorate or can remain without changes in time (Hojat et al., 2020). Although some research findings in this matter can be troublesome, most of them highlight a change in empathy, either positive as a result of implementing targeted interventions (Hojat, 2009; Hojat et al., 2013; Hegazi and Wilson, 2013; San-Martín et al., 2017b; Kataoka et al., 2019) or negative as a consequence of non-facilitating environments, negative clinical experiences, or lack of positive role models (Hojat et al., 2004, 2009; Neumann et al., 2011; San-Martín et al., 2017a). Furthermore, in the absence of targeted programs on empathy enhancement, family, and social and cultural environments have been described as external factors influencing the development of empathy (Hojat et al., 2004; Alcorta-Garza et al., 2016; Berduzco-Torres et al., 2020b), possibly due to the important role that social environments have on human relationships and social interactions (Täuber, 2018). On the other hand, findings from a large number of studies indicate that women are often more empathic than men, obtaining either higher scores on empathy measures (Guilera et al., 2019; Hojat et al., 2020) or better indicators in neurological measures related to empathy (Christov-Moore et al., 2014; Poynter, 2017; Toccaceli et al., 2018). Social learning, genetic predisposition, evolutionary underpinnings, and interpersonal styles have been described as possible explanations for such differences (Hojat et al., 2002a,b). Analysis of the neurobiological underpinnings of empathy reveals important quantitative gender differences in the basic networks involved in affective and cognitive forms of empathic responses.

### Medical Semiotics and the Promotion of Empathy Skills

A recent study comparing different medical curricula demonstrated that students matriculated in medical schools without a semiotics-based curriculum presented lower scores in empathy than the ones enrolled in medical schools in which a semiotic-based curriculum had been incorporated in their medical program (San-Martín et al., 2017b), reinforcing the idea of the potential importance that medical semiotics could have in the enhancement of empathy, especially in the early stages of medical studies. In accordance with this, different authors agree on the relevance that medical semiotics has in medicine (Nessa, 1996; Malterud, 2000; Kugelmann, 2003; Eriksen and Risør, 2014). Due to its role in the improving the interpretation along the entire clinical process, medical semiotics offers to clinicians a wider and more complete scenario to analyze their patients’ health conditions. This integrative scope includes not only biological, but also other factors that are influencing their patients’ health perceptions. Thus, this approach takes into consideration that human medicine is an enterprise in which the object of study is a human being, not a biological being only. In this regard, to understand human beings implies to understand human beings able to understand themselves and able to communicate this understanding. In consequence, this acknowledgment requires practitioners not only to have clinical knowledge, but also understanding abilities and interpersonal skills, all of which are necessary for establishing an optimal communication with the patient. In this sense, medical semiotics helps to narrow the gap of uncertainty and gives a more global understanding of the medical treatment process in which symptoms and clinical signs require an interpretation. All these characteristics possibly put semiotics as an important pedagogical tool for the early enhancement of empathy in medical and nursing students.

### Study Purpose

Based on the research framework previously described, the following hypothesis was tested: empathetic orientation of medical students, measured by the Jefferson Scale of Empathy (JSE), increases as a consequence of improving their capacity of understanding and expressing verbal and non-verbal communication in clinician-patient encounters. With this purpose, five goals were pursued: (i) to compare scores of the JSE by sex groups; (ii) to confirm if those scores present a significant improvement in time; (iii) to measure whether the improvement expected is exclusively for empathy or also occurs for inter-professional collaboration and lifelong learning abilities, the other two professional competencies that are also described as specific components of medical professionalism (Veloski and Hojat, 2006, p. 118) and that were used as internal controls in this study; (iv) to measure if students’ course scores obtained once the course was finished correlate with their global scores of the JSE (convergent validity); and (v) to determine if students’ course scores do not correlate with measures of inter-professional collaboration and lifelong learning (discriminant validity).

## Materials and Methods

### Participants and Design

The study, carried out in the Faculty of Medicine of the Universidad Privada Antenor Orrego (UPAO) of Trujillo (Peru), followed a quasi-experimental design. The study included two cohorts of third-year medical students who started a 4-month (17 weeks) course on medical semiotics. Of these, 269 students (92% of the entire number of students enrolled in both cohorts) agreed to participate.

**FIGURE 1:**
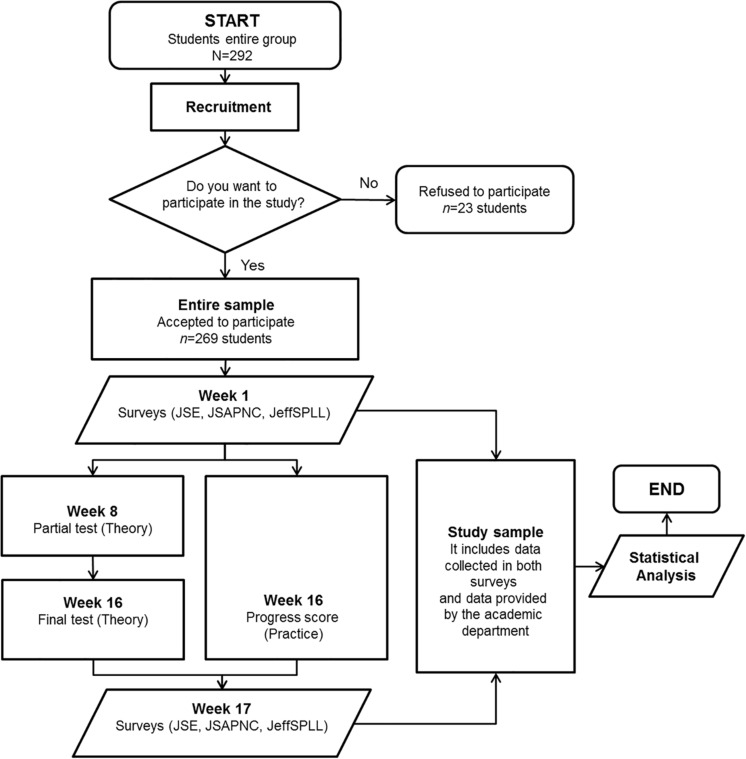
Overview of the workflow diagram of this study. JSE, Jefferson Scale of Empathy; JSAPNC, Jefferson Scale of Attitudes toward Physician-Nurse Collaboration; JeffSPLL, Jefferson Scale of Lifelong learning.

In the first week of classes in each semester (week 1), all students were invited to attend a workshop led by an external faculty who was part of the research team. During this activity, they were informed that this study was starting with the aim of measuring the development of three specific competencies associated with medical professionalism. In order to reduce possible bias in students’ participation derived from a social desirability, all students were informed that their participation was voluntary, anonymous and confidential and it was not related with their ordinary academic evaluation. Those who accepted to participate signed a written informed consent and received a multiple-choice questionnaire with specific psychometric measurements (see below). In the last week of classes (week 17), participants received a second questionnaire, identical to the first one. Both questionnaires were administered in paper and were returned in sealed envelopes. Participants included their “student code” (personal identifier) in each questionnaire they filled. In a later stage, this code was used to link both questionnaires with a data sheet brought by the academic department in charge of the course on medical semiotics. The data sheet included scores obtained by all students who were matriculated in the course. After this action was done, personal data were pseudo-anonymized replacing personal identifiers with a new identifier, making sure that research data belonging to the same student stayed together. A summary of the study design is shown in Figure 1.

An independent ethical committee for clinical research (Comité Ético de Investigación Clínica de La Rioja) approved the study design (Ref. CEICLAR PI 199) in Spain. The study was carried out in accordance with recommendations and authorization of the participating institution’s administration in Peru.

### Course on Medical Semiotics

The course on medical semiotics is mandatory and a core component of the medical curriculum of the Faculty of Medicine of the UPAO. The course is included in one of the two semesters of the third academic year of undergraduate medical studies. The course has a duration of four months (17 weeks) with an academic dedication of 28 weekly hours (17 credits) that are divided in: 5 weekly hours of theoretical classes, and 24 weekly hours of practical activities at the hospital. Together with this course, third-year medical students have to attend another two courses during the same academic semester: a course on “Diagnostic imaging and clinical laboratory,” with 8 weekly hours of practical activities (4 credits); and a course on “Pathophysiology,” with 4 weekly hours of theoretical and practical activities (3 credits).

Regarding the contents of the course on medical semiotics, the main objective of the course is that students are able to acquire abilities for interpreting symptoms and clinical signs in patients who attend clinic consultations. It is expected that students, at the end of the course, are able to identify patients’ general health problems, to reach a diagnosis, and to elaborate a plan of activities oriented to confirm a preliminary clinical diagnosis based on the patient’s interview (*anamnesis*) and medical history including the main group of pathologies. As part of their practice, students learn how to run a clinical interview and perform a physical examination. In addition, during the course students learn how to elaborate a clinical history of a patient who is in the hospital. Finally, students are trained on ethical and legal aspects related to patients’ rights. Students’ training is initially performed in settings that are different from the real ones through workshops, role-playing activities and clinical simulation stations. Later, abilities acquired are reinforced in clinical settings involving contact with real patients. During these visits, students perform a diagnosis that is later presented in class-room seminars. All activities performed are permanently mentored. A detailed explanation of the contents of the course is provided in the Supplementary Material. Finally, in the last years, the course has been reinforced by the acquisition of clinical simulation stations that are currently used as part of students’ training.

The students’ course evaluation is based on four scores: (i) a “partial test,” which evaluates theoretical contents provided in the first part of the course (week 1–7); (ii) a “final test,” which evaluates theoretical contents provided in the second part of the course (week 8–16); (iii) a “practical score,” which evaluates all practical activities performed along the entire course (week 1–16) that includes: class-room seminars, role playing activities, and rotations in a series of stations where typical clinical scenarios are represented; and (iv) a “final score,” which summarizes the scores obtained in the three aforementioned evaluations based on the following formula: FS = 0.55^∗^PS+0.20^∗^PT+0.25^∗^FT. According to the Peruvian educative system, the range of each score is from 0 to 20, a score equal or higher than 10.5 being considered as passed.

### Main Measures

Empathic orientation was measured using the medical students’ version of the Jefferson Scale of Empathy (JSE-S). The JSE-S is a psychometrically sound instrument developed specifically to measure empathy of medical students in the context of patient care (Hojat and Gonnella, 2015). The JSE S-Version includes 20 items answered following a Likert scale from 1 (*strongly disagree*) to 7 (*strongly agree*). The possible range of scores of the JSE is from 20 to 140. Higher scores indicate a higher empathic orientation. Three factors have been described as components of the Spanish medical students’ version of the JSE (Alcorta-Garza et al., 2005), distributed as follows: (i) “perspective taking,” with 10 worded items, which refers to the main component and the core ingredient of the empathy and the stepping-stone in empathic engagement; (ii) “compassionate care,” with seven worded items, which refers to the second main component; and (iii) “walking in the patient’s shoes” component that includes three worded items.

According to Hojat (2016), p. 109), the three underlying factors of the JSE are also supportive of the two pillars of the empathic engagement: the ability to develop an empathic understanding of the patient, commonly named seeing with the “mind’s eye”; and the ability to hear beyond the words spoken by the patient, also called the “third ear.” Based on the above-mentioned interpretation, the factors “perspective taking” and “walking in patients’ shoes” were grouped and used as a measure of “mind’s eye”; while “compassionate care” factor was used as a measure of “third ear.”

Inter-professional collaboration was measured using the Jefferson Scale of Attitudes toward Physician-Nurse Collaboration (JSAPNC). The JSAPNC was originally developed to address areas of physician-nurse interaction including authority, autonomy, shared responsibilities in patient care, collaborative decision making, and role expectations (Hojat et al., 1999), which are necessary in inter-professional collaborative work. The JSPANC is composed by 15 items that are answered following a Likert scale from 1 (*strongly disagree*) to 4 (*strongly agree*).

Physicians’ lifelong learning abilities were measured using the Jefferson Scale of Physicians Lifelong Learning (JeffSPLL-MS) (Wetzel et al., 2010). The JeffSPLL-MS measures the development of skills related to information gathering, the use of learning opportunities, and the self-motivation. The scale is composed by 14 items that are answered following a Likert scale from 1 (*strongly disagree*) to 4 (*strongly agree*).

Prior to using the three aforementioned instruments, authors obtained a written permission from Dr. Mohammadreza Hojat, from Thomas Jefferson University, for their use in this study. Satisfactory evidence supporting psychometric properties of these three instruments have been demonstrated in studies with medical students in Peru (Berduzco-Torres et al., 2020a,b; San-Martín et al., 2016).

Finally, information regarding age, sex, and the four students’ course scores (partial test, final test, practical score, and final score) was obtained in a pseudo-anonymized data sheet provided by the academic department.

### Statistical Analysis

The global score on the JSE was used as a measure of empathy. “Mind’s eye” and “third ear” constructs were used as measures of understanding and listening abilities that are part of the empathic engagement, respectively. The JSAPNC (inter-professional collaboration abilities) and the JeffSPLL (lifelong learning abilities), were used as internal controls and for criterion-related validity of evaluation tests of the medical-semiotics course. The reliability of the instruments administered was measured by the calculation of Cronbach’s alpha coefficient. This assessment was performed twice: at the beginning (week 1) and at the end (week 17) of the course. Following international recommendations (Frost et al., 2007), only measures with alpha coefficients equal or higher than 0.70 were included in the analyses.

Once the normality was studied, using Pearson’s chi-squared and Lilliefors-Kolmogorov-Smirnov tests, a non-parametric comparative analysis using *U* Mann-Whitney test was performed to compare differences in all measures according to gender, both at the beginning and at the end of the study. Furthermore, paired samples Wilcoxon test was used for comparing scores on measures at the beginning with the ones reported at the end. In those cases with a statistical significance confirmed (*p*-values lower than 0.05), effect size (*r*) was calculated following the formula described by Fritz et al. (2012) and Tomczak and Tomczak (2014) for non-parametric tests. The interpretation of the calculated *r*-value was similar as the one proposed by Cohen for Pearson’s correlation coefficient: a value equal to 0.50 is considered a large effect size with a crucial practical importance; equal to 0.30 is a medium effect size, with a moderate practical importance; and equal to 0.10 is a small effect size, with a negligible practical importance (Coolican, 2013, p. 395; Hojat and Xu, 2004). Finally, in correlation analyses Spearman’s coefficients were calculated. Those values were used: (i) to measure associations among empathy, inter-professional collaboration and lifelong learning scores; and (ii) to determine associations between empathy and academic evaluations (convergent validity) and between academic evaluations and scores of inter-professional collaboration and lifelong learning (divergent validity).

Data processing was carried out with R software, version 3.5.1 for Windows, and included the use of *nortest* (Gross et al., 2015), *multilevel* (Bliese, 2016), and *rstatix* (Kassambara, 2020) statistical packages.

## Results

### Participants

The entire sample of medical students who accepted to participate in the study and fully completed both questionnaires was composed by 86 male and 141 female students. Almost all students, with the exception of three, were Peruvians. According to place of origin, 65% were originally from Trujillo city, whereas the other 35% were from 30 different cities. The mean age was 22 years old with a range from 19 to 40 years (*SD* = 3).

### Reliability

The three psychometric instruments and the constructs “mind’s eye” and “third ear” showed acceptable reliability, given by Cronbach’s alpha coefficient, with values ranging between 0.70 and 0.85. The complete description of the scores of the instruments analyzed, for the whole sample, at the beginning and at the end of the study is reported in Table 1.

**TABLE 1:** Descriptive analysis and reliability of the main measures at the beginning and at the end of the study in the entire sample of medical students (*n* = 227).

**Main measures**	***n***	**PR**	**AR**	**Mdn**	***M* (*SD*)**	**Reliability**
**Beginning of the study**						
Empathy (JSE-S)	220	20–140	67–140	103	102 (16)	0.85
Mind’s eye	220	13–91	44–91	67	67 (10)	0.78
Third ear	224	7–49	7–49	36	35 (10)	0.83
Inter-professional collaboration (JSAPNC)	221	15–60	21–59	45	45 (6)	0.78
Lifelong learning (JeffSPLL-MS)	219	14–56	32–56	45	46 (5)	0.81
**End of the study**						
Empathy (JSE-S)	211	20–140	73–135	111	109 (14)	0.81
Mind’s eye	214	13–91	42–87	70	70 (9)	0.75
Third ear	221	7–49	14–49	41	40 (7)	0.70
Inter-professional collaboration (JSAPNC)	220	15–60	27–60	46	46 (5)	0.72
Lifelong learning (JeffSPLL-MS)	221	14–56	34–56	45	46 (5)	0.78

*JSE-S, Jefferson Scale of Empathy S-Version; JSAPNC, Jefferson Scale of Attitudes toward Physician-Nurse Collaboration; JeffSPLL-MS, MS-Version of the Jefferson Scale of Physicians Lifelong Learning; n, sample size; PR, possible range; AR, actual range; Mdn, median; M, mean; SD, standard deviation.*

### Sex Differences in Self-Reported Empathy

With regard to the first goal (differences on empathy by sex groups), female medical students reported higher global scores on empathy (*p* = 0.004; *r* = 0.20) and showed to be better listeners (*p* = 0.003; *r* = 0.20) than their male peers, but only at the beginning of the study. However, at the end of the study such differences had a negligible practical importance (*p* = 0.04; *r* = 0.14) or were not confirmed (*p* = 0.11), respectively. No differences by sex groups appeared for inter-professional collaboration and lifelong learning abilities, neither at the beginning nor at the end. The summary of these findings are reported in Table 2.

**TABLE 2:** Summary result of *U* Mann-Whitney tests comparing scores on main measures by sex groups, at the beginning and at the end of the study.

**Study groups**	**Start (week 1)**					**End (week 17)**				
	***n***	**Mdn**	***M* (*SD*)**	***p***	***r***	***n***	**Mdn**	***M* (*SD*)**	***p***	***r***
**JSE-S**										
Male group	84	95	98 (16)	0.004	0.20	78	108	107 (15)	0.04	0.14
Female group	136	106	104 (16)			133	112	111 (13)		
**Mind’s eye**										
Male group	84	66	65 (9)	0.10	–	79	69	68 (9)	0.04	0.14
Female group	136	67.5	68 (10)			135	71	71 (9)		
**Third ear**										
Male group	85	32	32 (10)	0.003	0.20	83	40	39 (7)	0.11	–
Female group	139	38	36 (10)			138	42	40 (7)		
**JSAPNC**										
Male group	84	45	45 (6)	0.24	–	84	46	45 (5)	0.07	–
Female group	137	46	46 (6)			136	47	47 (5)		
**JeffSPLL-MS**										
Male group	82	45	45 (5)	0.08	–	81	46	45 (5)	0.67	–
Female group	137	46	46 (5)			140	45	46 (5)		

*JSE-S, Jefferson Scale of Empathy S-Version; JSAPNC, Jefferson Scale of Attitudes toward Physician-Nurse Collaboration; JeffSPLL-MS, MS-Version of the Jefferson Scale of Physicians Lifelong Learning; n, sample size; Mdn, median; M, mean; SD, standard deviation; p, p-Value; r, effect size.*

### Enhancement of Empathy

**FIGURE 2:**
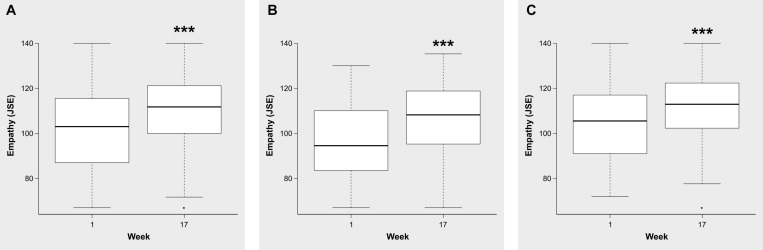
Comparison of the global scores on the JSE in the entire sample **(A)**, group of male **(B)** and female **(C)** medical students, between the beginning and the end of the course of medical semiotics. Midlines indicate the median, boxes indicate interquartile ranges, whiskers indicate the upper and lower adjacent values (within 1.5-fold the interquartile range), and isolated data points indicate outliers. ***p < 0.001.

With regard to the second goal (enhancement of empathy in time), comparisons between the beginning and the end showed a significant improvement in empathy’s global scores in the entire sample (*p* < 0.001) with a crucial practical importance (*r* = 0.45). This enhancement was confirmed separately in both sex groups, as is shown in Figure 2. In the entire sample, empathic understanding, measured by the “mind’s eye” construct (*p* < 0.001), and listening abilities, measured by the “third ear” construct (*p* < 0.001), also showed an important improvement at the end of the study with a moderate (*r* = 0.30) and crucial (*r* = 0.47) practical importance, respectively. When both sex groups were analyzed separately this enhancement was also observed, as it is shown in Table 3.

**TABLE 3:** Summary results of Wilcoxon signed-rank test for main measures by sex groups between the beginning (week 1) and the end (week 17) of attending a course on medical semiotics.

**Study groups**	**Diff.**	***p***	***r***
**JSE-S**			
Male group	+8	<0.001	0.51
Female group	+4	<0.001	0.45
**Mind’s eye**			
Male group	+1	0.03	0.24
Female group	+3	<0.001	0.30
**Third ear**			
Male group	+5.5	<0.001	0.58
Female group	+3	<0.001	0.39
**JSAPNC**			
Male group	+1	0.36	–
Female group	+0.5	0.04	0.16
**JeffSPLL-MS**			
Male group	+0.5	0.27	–
Female group	0	0.49	–

*JSE-S, Jefferson Scale of Empathy S-Version; JSAPNC, Jefferson Scale of Attitudes toward Physician-Nurse Collaboration; JeffSPLL-MS, MS-Version of the Jefferson Scale of Physicians Lifelong Learning; Diff., Median difference between the beginning (week 1) and the end (week 17) of the study according to the formula: Diff = Mdn_*week17*_ – Mdn_*week1*_; p, p-Value; r, effect size.*

The third goal was to determine whether the improvement of empathy during time was exclusively for empathy or it also affected inter-professional collaboration and lifelong learning abilities. Analyses revealed that only inter-professional collaboration abilities in the female students group showed a slight improvement in time (*p* = 0.04) but with a negligible practical importance (*r* = 0.16). No differences in time were observed in scores of inter-professional collaboration abilities in the male students group. In the case of lifelong learning abilities, no differences appeared in time, neither in the entire sample (*p* = 0.93) nor in male (*p* = 0.28) and female groups (*p* = 0.49). The summary of this analysis is also shown in Table 3.

### Correlation Analysis

A positive correlation was confirmed between empathy and inter-professional collaboration scores, at the beginning (ρ = +0.45; *p* < 0.001) and at the end (ρ = +0.37; *p* < 0.001). Empathy and lifelong learning scores also showed a positive correlation at the beginning (ρ = +0.25; *p* < 0.001) and at the end (ρ = +0.26; *p* < 0.001). Finally, inter-professional collaboration and lifelong learning were positively correlated at the beginning (ρ = +0.39; *p* < 0.001) and at the end (ρ = +0.20; *p* = 0.003). These findings confirmed that even these three elements are positively associated among them; the improvement observed in time was specific for empathy (third goal).

Finally, regarding the fourth and fifth goals (criterion validity of academic evaluation tests), a positive correlation was confirmed between empathy and the “practical score” (ρ = +0.16; *p* = 0.02). On the contrary, neither inter-professional collaboration (ρ = +0.07; *p* = 0.29) or lifelong learning scores (ρ = +0.06; *p* = 0.34) showed a correlation with “practical scores.” No correlations were either reported for the other two students’ course scores collected (“partial test,” “final test”) that evaluate theoretical contents of the course.

## Discussion

Cronbach’s alpha values, both at the beginning and at the end of the study, confirm an adequate reliability of the three psychometric instruments used in this study. These values were slightly higher than the ones originally reported in medical students in the United States (Hojat et al., 1999; Wetzel et al., 2010; Hojat and Gonnella, 2015) and similar than the ones previously reported in Peru (San-Martín et al., 2016; Berduzco-Torres et al., 2020a,b).

Slight differences in empathy and in listening abilities (associated with empathic engagement) between male and female medical students groups appeared at the beginning of this study, but not at the end. These findings are consistent with previous studies performed in Latin America: one in Dominican Republic, where medical semiotics, age and gender were described as contributors to empathy enhancement in medical students enrolled in pre-clinical phases of medical studies (San-Martín et al., 2017b); and another in Peru, where gender differences on empathy measures were also reported in undergraduate medical students (Berduzco-Torres et al., 2020b). These findings are also consistent with those reported in a number of studies carried out in the general population, where gender differences on empathy measures appear using different types of self-reported inventories (Guilera et al., 2019) and objective measures, such as a physiological reactions, brain activities, neurodevelopmental indicators, and genetic factors (Christov-Moore et al., 2014; Poynter, 2017; Toccaceli et al., 2018). On the other hand, the lack of gender differences in inter-professional collaboration and lifelong learning abilities observed in this study is also consistent with the different nature of these other two abilities (Hojat et al., 1999, 2009), in which gender apparently plays a negligible role of influence in comparison with other factors (Tuirán-Gutiérrez et al., 2019).

Correlation analyses, at the beginning and at the end of this study, confirm a positive relationship among empathy, inter-professional collaboration and lifelong learning measures, which is in accordance with their description as specific components of medical professionalism (Veloski and Hojat, 2006). However, the lack of improvement in inter-professional collaboration and lifelong learning abilities with time also confirms that the phenomenon measured in this study is specific for medical empathy. Taking into consideration three important aspects: (i) the training acquired during the medical semiotics course is mostly oriented to communication, listening and understanding abilities, which are necessary pre-requisites for establishing empathic encounters with the patients; (ii) the abovementioned abilities that trainees acquire are reinforced from different educative methodologies (i.e., role models, simulation-based methodologies, tutored visits to real clinical environments, workshops and discussing groups, narratives); and (iii) the greater academic dedication (28 weekly hours) that it requires, in comparison with the other two courses offered in the same semester (12 weekly hours), it is reasonable to consider that the improvement on empathy measures are mainly explained by the training received along the course. In fact these three aspects (targeted skills, different methodologies, and time of dedication) are in consonance with some characteristics described as components of effective empathy intervention in medical education (Fragkos and Crampton, 2020). Furthermore, this enhancement was observed in both sexes but with some minor differences: improvement on listening abilities (“third ear”) in male students presented the greatest effect size; on the other hand, understanding abilities (“mind’s eye”) in female students presented the greatest effect size. Such slight differences are probably related with certain gender differences associated with an empathic response.

Most important, findings observed in this experimental study confirm that empathy is amenable to change, a characteristic that has been previously discussed by some authors (Schumann et al., 2014; Hojat, 2016, p. 210). In this sense, the findings observed in this study confirm that medical semiotics offers another alternative for empathy enhancement that can be incorporated in medical education, similar to others, such as: narratives and video-taping methodologies (Suchman et al., 1997); training in interpersonal skills (Winefield and Chur-Hansen, 2000; Kaplan-Liss et al., 2018); tutored rotations in people-oriented clinical services (Elizur and Rosenheim, 1982); training with simulation-based methodologies (Elizur and Rosenheim, 1982; Abdool et al., 2017); or social volunteering (Sin et al., 2019).

Finally, from all academic evaluations collected, only the summary score of practical activities (those that are behavioral and attitudinal) showed a positive correlation with the global scores of empathy, proving a convergent validity. Conversely, a lack of significant relationship between inter-professional collaboration and lifelong learning scores and the practical evaluation’s scores (discriminant validity) reinforces the fact that competencies acquired during the medical semiotics course are specifically relevant for the improvement of students’ empathic abilities. On the other hand, the lack of correlation between empathy and theoretical evaluations is consistent with the contents of the course, where students acquire not only behavioral abilities, also medical knowledge that is necessary for reaching a medical diagnosis.

In conclusion, these findings offer empirical evidence supporting the amenability of empathy, as a professional competence, to change with an adequate training. It also remarks the positive effect that a targeted methodology focused on communication skills and understanding abilities in clinical settings has on the enhancement of empathy in medical students. However, more empirical research is still needed to determine whether this effect persists in the long-term when medical students reach advanced courses and in clinic internships.

### Limitations

The study followed a quasi-experimental design (also called non-randomized or pre-post intervention study). This type of design is often used in the literature to evaluate the benefits of a specific intervention and when it is not logistically feasible or ethical to conduct a randomized controlled trial (Harris et al., 2004). However, authors are aware that quasi-experimental designs have some limitations in comparison with experimental designs with regard to assessing causality (i.e., lack of control group). Furthermore, there are three medical schools in Trujillo with big differences among them, not only in programs, but also in the facilities, evaluation processes and students’ profile. This study was performed in one of the two private medical schools located in Trujillo city. It is possible that differences in number of students or group sizes (group of small classes vs. group of big classes) may play a role and influence the other two medical schools. However, the study of this effect may open new areas of research, where the different curricula between the public and private medical schools may also be compared.

## Data Availability Statement

The raw data supporting the conclusions of this article will be made available by the authors, without undue reservation, to any qualified researcher.

## Ethics Statement

The studies involving human participants were reviewed and approved by the Comité Ético de Investigación Clínica de La Rioja (Ref. CEICLAR PI 199). The participants provided their written informed consent to participate in this study.

## Author Contributions

LV was in charge of the study’s overall design. LF-R and VB-Z were in charge of coordination with the management department in the participating institution, students’ recruitment, and data collection. MS-M and LV performed the statistical processing of data. LV and RD were in charge of drafting the manuscript. All authors contributed during the interpretation process of the results, and approved the final manuscript.

## Conflict of Interest

LF-R and VB-Z were faculty members of the university where this study was performed. The remaining authors declare that the research was conducted in the absence of any commercial or financial relationships that could be construed as a potential conflict of interest.
