# Supplementary material for: Empathy Enhancement Based on a Semiotics Training Program: A Longitudinal Study in Peruvian Medical Students
Source: Front Psychol. 2020 Oct 29;11:567663. doi: 10.3389/fpsyg.2020.567663 (PMC7658416; doi:10.3389/fpsyg.2020.567663)
Supplement: Supplementary file 1 [file Table_1.pdf]

# SUPPLEMENTARY MATERIAL

**Contents of the course on medical semiotics by weeks: Description of the procedural and attitudinal contents the student or attendee of the course will fulfil**

| Week | Procedural contents | Attitudinal Contents |
|---|---|---|
| 1 | The attendee knows and applies the theoretical aspects of clinical questioning correctly using the semiological technique that will allow him/her to obtain the most information possible on the parentage, patient profile and current disease. | The attendee shows respect for the patient's personality and dignity. |
| 2 | The attendee knows and applies the theoretical aspects of clinical interrogation correctly using the semiological technique that will allow him/her to obtain the most information possible on family and pathological history and allow a review by organs and systems. | The attendee shows respect during the clinical examination of the patient. |
| 3 | The attendee demonstrates and applies correct techniques for taking blood pressure, pulse, temperature, respiratory rate, weight, height, and body mass index. Also, the attendee correctly describes the general assessment and mental examination of a patient. | The attendee shows respect, confidentiality in the clinical examination. |
| 4 | The attendee properly examines skin, subcutaneous cellular tissue, and lymph nodes.<br><br>The attendee performs a basic ophthalmological examination, including: visual acuity, external examination, pupils, ocular motility, and fundus. The attendee uses the ophthalmoscope correctly.<br><br>The attendee performs an adequate examination of the nose and ears (eardrum membrane by otoscopy and nasal mucosa by rhinoscopy). | The attendee recognizes the importance of the examination and its application in the diagnostic process. |
| 5 | The attendee performs proper mouth and pharynx examinations, as well as corrects palpation of the thyroid by anterior and posterior methods.<br><br>The attendee sequentially and correctly examines the respiratory system, knowing how to differentiate the normal findings upon inspection, auscultation (normal respiratory sounds), palpation (vocal vibration) and percussion (loudness).<br><br>The attendee sequentially and correctly examines the | The attendee recognizes the importance of the clinical examination and its application in the diagnostic process. |

| | | |
|---|---|---|
| | cardiovascular system, locating the apical impulse, auscultatory foci, S1 and S2 heart sounds, normal pulse characteristics, and measures jugular venous pressure and interprets it. | |
| 6 | The attendee correctly examines the abdomen, specifying the regions and organs. The attendee maintains the sequence of recommended techniques.<br><br>The attendee properly palpates the liver, (hepatic height), spleen. The attendee performs the digital rectal examination and correctly identifies the structures (prostate, rectum, and anus).<br><br>The attendee correctly evaluates the Kidneys, ureters, bladder, male and female genitalia.<br><br>The attendee correctly evaluates the musculoskeletal system, palpation, joint range, muscle strength and the most important manoeuvres. | The attendee recognizes the importance of the clinical examination and expresses respect in the diagnostic process. |
| 7 | The attendee correctly evaluates the musculoskeletal system: inspection, palpation, joint range, muscle strength and most important manoeuvres.<br><br>The attendee performs a complete neurological exam that includes consciousness, motor and sensory function, meningeal signs.<br><br>The attendee performs a complete neurological exam that includes cranial nerves. | The attendee recognizes the importance and expresses respect for the examination and its application in the diagnostic process. |
| **8** | **Partial test** | |
| 9 | The attendee performs the anamnesis and physical examination of patients who will be assigned by role of health problems: pain and fever. The attendee drafts reports including anamnesis, physical examination, summary, health problems, diagnostic hypotheses and diagnostic plan. | The attendee assumes with analysis the steps of the problem-oriented medical history approach. |
| 10 | The attendee performs the anamnesis and physical examination of the patients who will be assigned according to the role of health problems: arterial hypertension, shock and edema.<br><br>The attendee writes a report that will include anamnesis, physical examination, summary, health problems, diagnostic hypotheses and diagnostic plan. | The attendee demonstrates critical judgment in the statement of the hypothesis and diagnostic plan. |

| | | |
|---|---|---|
| 11 | The attendee performs the anamnesis and physical examination of patients who will be assigned by role of health problems: dyspnea, respiratory failure cough, hemoptysis, and murmurs heart sounds.<br><br>The attendee writes a report that will include the anamnesis, physical examination, summary, health problems, diagnostic hypotheses and diagnostic plan. | The attendee demonstrates perseverance and training in the steps of physical examination and clinical judgment. |
| 12 | The attendee performs the anamnesis and physical examination of the patients assigned to them according to the role of health problems: chest pain, abdominal pain and hepatorenal syndrome.<br><br>The attendee writes a report that will include the anamnesis, physical examination, summary, health problems, diagnostic hypotheses and diagnostic plan. | The attendee assumes with dedication the task and adequately plans the methodology of approach to the patient for the presentation of diagnostic assumptions. |
| 13 | The attendee performs the anamnesis and physical examination of patients who will be assigned by role of health problems: gastrointestinal bleeding, diarrhea, and jaundice.<br><br>The attendee writes a report that will include the anamnesis, physical examination, summary, health problems, diagnostic hypotheses and diagnostic plan. | The attendee is responsible for following the sequences of the semiological methodology. |
| 14 | The attendee performs the anamnesis and physical examination of the patients assigned to them according to the role of health problems: acid balance - base, headache, urinary system alteration, joint pain, pyramidal and extrapyramidal syndromes.<br><br>The attendee writes a report that will include the anamnesis, physical examination, summary, health problems, diagnostic hypotheses and diagnostic plan. | The attendee is responsible for the foundation of the diagnostic impression and differential diagnoses. |
| 15 | The attendee performs the anamnesis and physical examination of patients who will be assigned by role of health problems: anemia, vertiginous syndrome, neuropathy and myopathy, acute confusional syndrome, and threatening emergencies.<br><br>The attendee writes a report that will include the anamnesis, physical examination, summary, health problems, diagnostic hypotheses and diagnostic plan. | The attendee timely differentiates an urgent and emergency situation in his/her clinical practice. |
| **16** | **Structured objective clinical examination / Final Exam** | |

| | |
|---|---|
| **17** | **Substitutory Exam** |
